# Salivary Gland Proteome during Adult Development and after Blood Feeding of Female *Anopheles dissidens* Mosquitoes (Diptera: Culicidae)

**DOI:** 10.1371/journal.pone.0163810

**Published:** 2016-09-26

**Authors:** Benjarat Phattanawiboon, Narissara Jariyapan, Chonlada Mano, Sittiruk Roytrakul, Atchara Paemanee, Sriwatapron Sor-Suwan, Patchara Sriwichai, Atiporn Saeung, Paul A. Bates

**Affiliations:** 1 Department of Parasitology, Faculty of Medicine, Chiang Mai University, Chiang Mai, Thailand; 2 National Center for Genetic Engineering and Biotechnology (BIOTEC), National Science and Technology Development Agency, Pathumthani, Thailand; 3 Department of Medical Entomology, Faculty of Tropical Medicine, Mahidol University, Bangkok, Thailand; 4 Division of Biomedical and Life Sciences, Faculty of Health and Medicine, Lancaster University, United Kingdom; University of Heidelberg Medical School, GERMANY

## Abstract

Understanding changes in mosquito salivary proteins during the time that sporozoite maturation occurs and after blood feeding may give information regarding the roles of salivary proteins during the malarial transmission. *Anopheles dissidens* (formerly *Anopheles barbirostris* species A1) is a potential vector of *Plasmodium vivax* in Thailand. In this study, analyses of the proteomic profiles of female *An*. *dissidens* salivary glands during adult development and after blood feeding were carried out using two-dimensional gel electrophoresis coupled with nano-liquid chromatography-mass spectrometry. Results showed at least 17 major salivary gland proteins present from day one to day 21 post emergence at 8 different time points sampled. Although there was variation observed, the patterns of protein expression could be placed into one of four groups. Fifteen protein spots showed significant depletion after blood feeding with the percentages of the amount of depletion ranging from 8.5% to 68.11%. The overall results identified various proteins, including a putative mucin-like protein, an anti-platelet protein, a long form D7 salivary protein, a putative gVAG protein precursor, a D7-related 3.2 protein, gSG7 salivary proteins, and a gSG6 protein. These results allow better understanding of the changes of the salivary proteins during the adult mosquito development. They also provide candidate proteins to investigate any possible link or not between sporozoite maturation, or survival of skin stage sporozoites, and salivary proteins.

## Introduction

In *Anopheles* mosquitos, salivary glands are crucial organs for the development and transmission of *Plasmodium* parasites. The parasites must invade and reside for some period of time in the salivary glands to enable maturation and effective transmission [1, 2]. When the infected mosquito takes the next blood meal, the mature sporozoites are mixed with the saliva and injected into the new host with the bite. In general, the extrinsic incubation period for malaria parasites in the mosquito vector is approximately 9 to 21 days depending on the species of both the mosquitoes and parasites and also the ambient temperature [3, 4, 5, 6]. Understanding the changes in mosquito salivary proteins after the blood feeding and during the time that the sporozoite maturation occurs may give greater information regarding the role of salivary proteins during malarial transmission.

Changes in the amounts of saliva and its composition depend on the physiological state of the insects. In another insect disease vector, phlebotomine sand flies, differences in the amount, composition and enzyme activity of salivary proteins are found according to species, age and diet [7]. In various mosquito species, several studies have demonstrated changes in the total amount of salivary gland proteins and/or the salivary gland protein expression in sugar feeding and blood feeding mosquitoes, for example, *Aedes aegypti* [8, 9], *Anopheles gambiae* [10, 11], *Anopheles barbirostris* species A2 [12], *Anopheles camprestris*-like [13], *Armigeres subalbatus* [14], and *Mansonia uniformis* [15]. However, there has been little work on the proteome of mosquito salivary glands at different time-points in adult development.

In Thailand, *Anopheles dissidens* (formerly *Anopheles barbirostris* species A1) [16, 17] is a potential vector for *Plasmodium vivax* [18]. However, to date only the ultrastructural morphology and sodium dodecyl sulphate polyacrylamide gel electrophoresis (SDS-PAGE) profile of female salivary gland proteins of *An*. *dissidens* have been reported [19, 20]. In addition, there is no information on any changes in salivary gland proteins after blood feeding in this mosquito species. Therefore, in this current study the salivary gland proteome of female *An*. *dissidens* was analyzed during adult development and also after blood feeding, using two-dimensional gel electrophoresis (2-DE) coupled with nano-liquid chromatography-mass spectrometry (nanoLC-MS). The results showed the expression of salivary gland proteins in the mosquitoes aged from 0 to 21 days varied, and also were significantly depleted in protein level after blood feeding.

## Materials and Methods

### Mosquitoes

A colony of *An*. *dissidens* maintained for many consecutive generations in an insectary at the Department of Parasitology, Faculty of Medicine, Chiang Mai University, Thailand was used in this study. The methods for rearing mosquitoes described by Kim *et al*. [21] were used. For blood feeding, adult male albino rats, *Rattus norvegicus*, obtained from a breeding colony in the laboratory animal house, Faculty of Medicine, Chiang Mai University were used in this study.

### Ethical clearance

The protocols were approved by the Animal Ethics Committee of the Faculty of Medicine, Chiang Mai University, Chiang Mai, Thailand (Protocol Number 05/2558).

### Salivary gland dissection

In the developmental experiments, female mosquitoes were collected at 0, 1, 2, 3, 8, 12, 16 and 21 days after emergence. Collections on day 0 were made within two hours of emergence and on other days within the same two hour window. Therefore, day 0 = 0–2 hours after emergence, day 1 = 24–26 hours, day 2 = 48–50 hours, day 3 = 72–74 hours, day 8 = 192–194 hours, day 12 = 288–290 hours, day 16 = 384–386 hours, and day 21 = 504–506 hours after emergence. The females were cold anaesthetized on ice before being dissected to remove the salivary glands. Salivary glands were dissected in phosphate-buffered saline [10 mM NaH2PO4, 145 mM NaCl (pH 7.2)] and carefully transferred to a microcentrifuge tube in a small volume of phosphate-buffered saline. The samples were kept at -80°C until use. Salivary gland proteins were quantified using a Micro BCA Protein Assay Kit (Pierce, USA). Eighty individuals were used for each experiment, equivalent to approximately 88 μg of total protein, as the total salivary gland protein content of each female mosquito at day 2 post emergence was on average 1.10 ± 0.05 μg/gland pair. For the blood feeding experiment, 12 to 14 day old sugar-fed mosquitoes were allowed to feed on blood from immobilized rats and those that had fed to repletion were dissected immediately to remove the salivary glands as described above. Sugar-fed mosquitoes from the same cohort were used as a control. Both development and blood feeding experiments were each performed three times on different cohorts of mosquitoes, and 2D gel analysis performed at all sampling points for each of these biological replicates.

### Two-dimensional gel electrophoresis

Two-dimensional gel electrophoresis was performed using a 2D system from GE Healthcare, UK. The extracted salivary glands were desalted using a 2-D Clean-Up kit (GE Healthcare, UK). Each sample was solubilized in a 125 ml sample solubilization solution (8 M urea, 50 mM DTT, 4% CHAPS, 0.2% 3/10 Bio-lyte Ampholyte, 0.002% Bromophenol Blue) and then loaded onto an IPG strip (pI 3–10, 7 cm, GE Healthcare, UK) to perform the first dimension isoelectric focusing separation. The IPG strips were the transferred to an Ettan IPGphor III (GE Healthcare, UK) apparatus, which was operated at 50 μA/strip and the default cell temperature of 20°C. The focusing conditions utilised a program of progressively increasing voltage: 300 V for 1 hour; 1000 for 1 hour; 5000 V for 1 hour; and 7600 Vh^− 1^. Then, the focused IPG strips were incubated in 10 ml sodium dodecyl sulphate equilibration buffer (6 M urea, 2% SDS, 0.05 M Tris, pH 8.8, 30% glycerol, 0.002% Bromophenol Blue) containing 100 mg dithiothreitol for 15 minutes and then for a further 15 minutes in five ml of equilibration buffer containing 125 mg iodoacetamide. The equilibrated strips were applied to the surface of vertical 15% sodium dodecyl sulphate polyacrylamide gels and proteins separated in the second dimension using the Mini-PROTEAN Tetra Electrophoresis System (Bio-Rad, USA). Protein molecular weight markers (broad range, Bio-Rad, USA) were applied to each gel.

### Coomassie Brilliant Blue staining and gel image analysis

Following electrophoresis, the gels were stained with Coomassie Brilliant Blue. First, the gels were fixed in 50% methanol and 10% acetic acid for 30 minutes, then stained with 1% Coomassie Brilliant Blue in 10% methanol and 5% acetic acid for two hours, and finally de-stained in 10% methanol and 5% acetic acid until dark protein bands were visible. The gels were scanned using the ImageScanner III (GE Healthcare, UK). A bioinformatics program, Image Master 2D Platinum 7.0 (GE Healthcare, UK) was used to detect the number of spots in each gel and their molecular weight and isoelectric point, and measure the expression volume of each spot.

### Protein quantification and statistical analysis

Three independent biological replicated 2-DE gel images were analyzed using the Image Master 2D Platinum 7.0 software (GE Healthcare, UK). This program was used to measure all protein spot densities. Heat shock cognate (HSC) 70 was used as an internal control protein to normalize quantitation. Previous work showed no change in HSC70 expression in *Ae*. *aegypti* salivary glands in response to heat shock [22] and blood feeding [8], and in *An*. *barbirostris* species A2 salivary glands in response to ageing [12]. The choice of HSC70 in the current study was further validated by analysis of three-dimensional images of HSC70 peaks (S1 Fig), showing stable levels of expression in *An*. *dissidens* on various different days of development. Normalisation was performed using the Image Master 2D Platinum 7.0 software using a spot volume of 0.02 for HSC70.

For developmental experiments, the mean and standard deviations were calculated using SPSS version 22.0 software. The analysis of variance (One Way ANOVA) was used to detect significant differences of the densities of the same spot in triplicate gels. Post Hoc analysis (Duncan’s Multiple Range test) was used to test the statistical difference of the means of the expression level between all age groups, and in figures and tables the use of different letters (a, b, c, d, e, f) indicates significantly different levels of protein expression (*p* < 0.05), i.e. groups labelled with the letter “a” are not significantly different from each other, but are different to groups labelled with b, c, d, e, or f, and so on. Any given letter is only relevant within that protein (i.e. “a” in SN1 has nothing to do with “a” in SN2). In the blood feeding experiment, the protein expression level was assessed using a Student’s t-test, *p*<0.05.

### In-gel digestion

Protein spots of interest were excised from the two-dimensional electrophoresis gels using sterile surgical blades ensuring that techniques were aseptic. The gel pieces were subjected to in-gel digestion using an in-house method developed by Proteomics Laboratory, National Center for Genetic Engineering and Biotechnology, National Science and Technology Development Agency, Thailand [23]. The gel plugs were dehydrated using 100% acetonitrile, reduced with 10 mM dithiothreitol in 10 mM ammonium bicarbonate at room temperature for one hour and alkylated at room temperature for one hour in the dark in the presence of 100 mM iodoacetamide in 10 mM ammonium bicarbonate. After alkylation, the gel pieces were dehydrated twice using 100% acetonitrile for 5 minutes. To perform in-gel digestion of proteins, 10 μl of trypsin solution (10 ng/μl trypsin in 50% acetonitrile /10 mM ammonium bicarbonate) was added to the gels followed by incubation at room temperature for 20 minutes, and then 20 μl of 30% acetonitrile was added to keep the gels immersed throughout digestion. The gels were incubated at 37°C for a few hours or overnight. To extract peptide digestion products, 30 μl of 50% acetonitrile in 0.1% formic acid was added to the gels, and then the gels were incubated at room temperature for ten minutes in a shaker. Peptides extracted were collected and pooled together in a new tube. The pool extracted peptides were dried using a vacuum centrifuge and kept at -80°C for further mass spectrometric analysis.

### NanoLC-MS analysis and protein identification

The digested protein was injected into an Ultimate 3000 LC System (Dionex, CA) coupled to an ESI-Ion Trap MS (HCT Ultra PTM Discovery System, Bruker, Germany) with electrospray at a flow rate of 300 nl/min to a nanocolumn (Acclaim PepMap 100 C18, 3 μm, 100A, 75 μm id x 150 mm). A solvent gradient (solvent A: 0.1% formic acid in water and solvent B: 80% 0.1% formic acid in 80% acetonitrile) was run for 40 minutes. Mascot from Matrix Science Ltd. (London, UK) was used to search all of the tandem mass spectra [24]. The resulting sequences were searched against the National Center for Biotechnology nonredundant (NCBInr) protein database. The searching parameters were set as follows: enzyme of specificity strict trypsin, one missed cleavage, fixed modifications of Carbamidomethyl (C), oxidation (Met), peptide tolerance of 100 ppm, Fragment Mass Tolerance of ±0.5 Da, peptide change of 1+, and monoisotopic. Protein identification was made on the basis of a statistically significant Mowse score (≥ 30). All accession numbers of the best match proteins presented in this study are available online at http://www.ncbi.nlm.nih.gov.

## Results

### Proteomic analysis of *An*. *dissidens* female salivary glands after emergence

**Fig 1:**
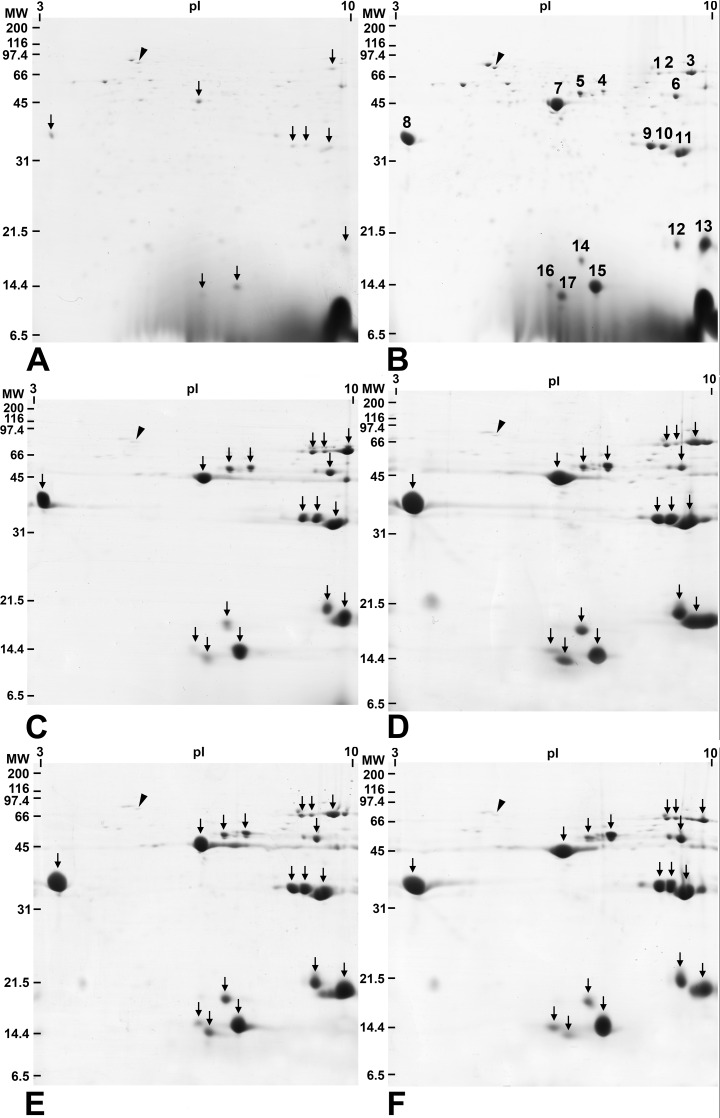
Representative 2-DE gels of salivary gland proteins extracted from 80 female mosquitoes at different ages. (A) 0 day, (B) 1 days, (C) 3 days, (D) 12 days, (E) 16 days, and (F) 21 days old. Molecular mass markers are indicated on the left in kDa. Isoelectric points (pI) are indicated at the top. Arrows indicate major salivary gland proteins. Arrowhead indicates an internal control protein. Numbers are corresponding to the major salivary gland proteins in Table 1. The large spot at the base of the right side gels of Fig 1A and B is an artifact of separation. It was seen on the three gels shown with these samples only.

Two-dimensional electrophoresis gels of salivary gland samples collected 0, 1, 3, 12, 16 and 21 days after emergence are shown in Fig 1A–1F. The proteins accumulated following emergence, to reach a typical profile after 1 day (Fig 1B). The profile consisted of approximately 80 well-resolved spots with molecular masses of 13 to 80 kilodaltons and isoelectric points ranging from 3.8 to 10. Of these, there were 17 major salivary gland proteins observed from 1 to 21 days after emergence for which peptide sequence data was obtained (Table 1). These were each given a spot number (SN) from 1 to 17 (Table 1, Fig 1B). In newly emerged mosquitoes (day 0), the 2D profile revealed 9 of the 17 major protein spots, namely spot numbers SN3, 7–11, 13, 15 and 17 (Fig 1A). The remaining 8 major spots were first detected in the salivary glands of females on day 1 after emergence. Each of the 17 major protein spots were excised and subjected to nanoLC-MS for identification. The results of mass spectrometry analysis are summarized in Table 1. The 17 major protein spots gave significant matches to protein sequences from various species of mosquito. The identified proteins included three apyrases (SN 1–3), four putative-mucin like proteins (SN 4–7), an anti-platelet protein (SN 8), two long form D7 proteins (SN 9 and 10), a gVAG precursor (SN 11), three short form D7 related proteins (SN 12–14), two gSG7 proteins (SN 15 and 16) and gSG6 (SN 17).

**Table 1:** Details of 17 major protein spots identified by nano-liquid chromatography-mass spectrometry in the female salivary glands of *An*. *dissidens*.

SN^a^	Accession number^b^, Protein description [species]	Protein score^c^	No. of peptides/ % coverage	Database MW^d^/ pI^e^	Observed MW/ pI	Peptide sequence(s)
**1**	CAB40345, apyrase [*Anopheles gambiae*]	65	1/1	62.08/ 8.88	68/ 8.8	R.VFHTVQELR.K
**2**	CAB40345, apyrase [*Anopheles gambiae*]	57	1/1	62.08/ 8.88	68/ 8.9	R.VFHTVQELR.K
**3**	AAO06829, salivary apyrase [*Anopheles stephensi*]	50	1/1	64.70/ 6.77	68/ 9.7	R.LTVYFDQR.G
**4**	AAL85611, putative mucin-like protein [*Aedes aegypti*]	35	1/3	28.96/ 5.10	56/ 7.6	M.AVTISHSK.V
**5**	AAL85611, putative mucin-like protein [*Aedes aegypti*]	35	1/3	28.96/ 5.10	55/ 7.2	M.AVTISHSK.V
**6**	AAL85611, putative mucin-like protein [*Aedes aegypti*]	35	1/3	28.96/ 5.10	54/ 9.3	M.AVTISHSK.V
**7**	AAL85611, putative mucin-like protein [*Aedes aegypti*]	35	1/3	28.96/ 5.10	48/ 6.8	M.AVTISHSK.V
**8**	KFB41046, anti-platelet protein [*Anopheles sinensis*]	212	4/18	26.78/ 4.12	38/ 4	R.LMNPTIDLVNTIEK.Y K.DVQGLVKESEK.S R.ELDEGLIDR.E R.EQELSDCIVDKR.D
**9**	KFB42863, long form D7 salivary protein [*Anopheles sinensis*]	180	5/15	35.63/ 8.35	35/ 8.7	K.VYAADPSIK.K K.KGESYFAYCEK.R R.QYELTGSAQLK.D K.DSIDCIFR.G R.SANYAYLVLGK.V
**10**	KFB42863, long form D7 salivary protein [*Anopheles sinensis*]	254	5/15	35.63/ 8.35	34/ 9	K.VYAADPSIK.K K.KGESYFAYCEK.R R.QYELTGSAQLK.D K.DSIDCIFR.G R.SANYAYLVLGK.V
**11**	KFB54026, putative gVAG protein precursor [*Anopheles sinensis*]	117	2/7	31.51/ 9.31	33/ 9.4	K.QFPYAGQNIAITK.Y K.VGCSLWYWK.D
**12**	AAL16039, short form D7r1 salivary protein [*Anopheles arabiensis*]	43	1/6	19.04/ 9.17	20/ 9.3	K.LIKPLNAIEK.D
**13**	ACR54288, a348 (D7 related) [*Anopheles anthropophagus*]	389	6/27	18.96/ 9.34	19/ 9.8	K.TIGFVDK.D K.TIGFVDKDGR.G K.LIKPLNAIEK.D K.CMLQSNSAESFK.K K.CMLQSNSAESFKK.V K.VFDLTELVLAGK.L
**14**	KFB42861, D7-related 3.2 protein [*Anopheles sinensis*]	142	2/5	18.68/8.12	18/ 7.2	K.KAVDYVELLR.A K.AVDYVELLR.A
**15**	KFB36874, gSG7 salivary protein [*Anopheles sinensis*]	130	2/8	16.79/5.94	16/ 7.3	K.YGVQVQLR.E K.YGVQVQLREPLVK.K
**16**	KFB36874, gSG7 salivary protein [*Anopheles sinensis*]	68	1/5	16.79/5.94	14/ 6.6	K.YGVQVQLR.E
**17**	CAC35522, gSG6 protein [*Anopheles gambiae*]	50	1/8	13.66/ 5.3	12 / 6.8	K.QKQWIDR.D
**Control**	ABF18332, heat shock cognate 70 [*Aedes aegypti*]	316	7/11	71.4/5.3	77.0/5.4	R.TTPSYVAFTDTER.L K.NQVAMNPTNTIFDAK.R K.DAGTISGLNVLR.I R.IINEPTAAAIAYGLDK.K R.IINEPTAAAIAYGLDKK.T K.LLQDFFNGK.E K.FELSGIPPAPR.G

^a^Spot number refers to those shown in Fig 1B

^b^Accession number of the best hit of proteins from mosquitoes and/or arthropod species

^c^Mowse score ≥ 30

^d^MW: molecular mass

^e^pI: isoelectric point

**Fig 2:**
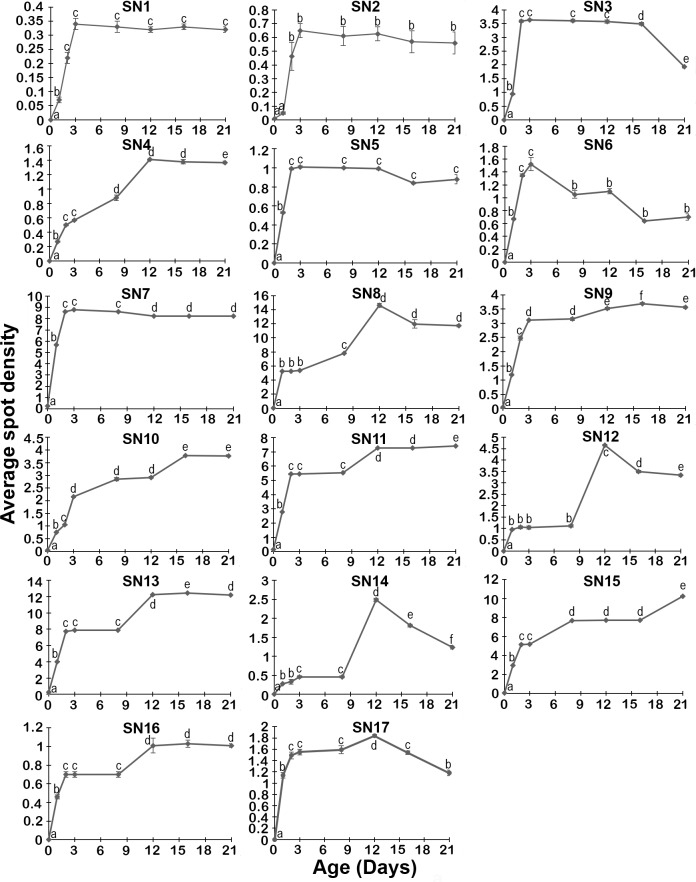
Expression levels of the 17 major protein spots determined at different ages in adult developmental time points. The y-axis represents relative expression level normalized with heat shock cognate (HSC) 70, and the x-axis represents different ages on days 0, 1, 2, 3, 8, 12, 16, and 21, accordingly. Different letters (a, b, c, d, e, f) indicate significantly different levels of protein expression (*p* < 0.05), i.e. groups labelled with one letter (e.g. “a”) are not significantly different from each other, but are different to groups labelled with a different letter (e.g. b, c, d, e, or f), and so on. Any given letter is only relevant within that protein (i.e. “a” in SN1 has nothing to do with “a” in SN2). Error bars are plotted for all points, but are too small to be visualised in some cases.

The relative expression levels of the 17 major protein spots determined at different ages in adult development are shown in Fig 2 and S1 Table. The 17 protein spots varied in their expression across the 8 time points sampled (0, 1, 2, 3, 8, 12, 16 and 21 days). Although there was variation observed, the patterns of expression could be placed into four groups. The largest (Group 1) was represented by SN4, 10, 11, 13, 15 and 16, which tended to show a more or less steady increase in expression with age of mosquito, but reaching a plateau at the latter time points. Another large group (Group 2) consisted of SN1, 2, 5, 7, 9 and probably SN3, in which the relative expression climbed rapidly to day 3, and then remained more or less constant for the remaining time points, except SN3 that dropped off at the last time point. The third group (Group 3) included SN8, 12, 14 and 17, which showed an early steep rise, a levelling off, then a peak on day 12, and decreased expression thereafter. Finally, there was SN6 (Group 4), which showed a unique expression pattern, rising to peak at day 3, and then steadily falling thereafter. A homologue of a heat shock cognate 70 kDa protein from *Ae*. *aegypti* (accession number ABF18332) was used as an internal control in the 2-DE gels (Tables 1 and 2, Figs 1 and 2). This control protein is indicated by an arrowhead in each panel of Fig 1 and showed no significant difference in density between samples.

**Table 2:** Amounts of depletion of the major salivary gland proteins after blood feeding of *An*. *dissidens* mosquitoes.

SN^a^	ASD ± SD^b^		Amount depleted	% depletion
	Sugar fed	Blood fed		
**1**	0.45 ± 0.02	0.34 ± 0.02	0.11 ± 0.04	25.41^c^
**2**	0.90 ± 0.03	0.88 ± 0.02	0.02 ± 0.04	1.86
**3**	4.55 ± 0.01	3.02 ± 0.01	1.53 ± 0.01	33.65^c^
**4**	0.98 ± 0.02	0.55 ± 0.02	0.42 ± 0.03	43.29^c^
**5**	1.63 ± 0.03	0.52 ± 0.03	1.11 ± 0.00	68.11^c^
**6**	1.11 ± 0.02	1.01 ± 0.03	0.09 ± 0.04	8.15^c^
**7**	10.86 ± 0.02	5.52 ± 0.02	5.35 ± 0.02	49.21^c^
**8**	11.23 ± 0.02	5.86 ± 0.02	5.37 ± 0.01	47.82^c^
**9**	4.01 ± 0.04	2.30 ± 0.03	1.71 ±0.02	42.57^c^
**10**	3.60 ± 0.03	1.92 ± 0.02	1.68 ± 0.04	46.63^c^
**11**	7.94 ± 0.02	5.31 ± 0.03	2.62 ± 0.02	33.07^c^
**12**	3.06 ± 0.02	3.02 ± 0.02	0.04 ± 0.04	1.25
**13**	12.55 ± 0.03	9.67 ± 0.03	2.88 ± 0.02	22.92^c^
**14**	2.08 ± 0.02	1.08 ± 0.02	1.00 ± 0.02	48.15^c^
**15**	6.71 ± 0.02	4.12 ± 0.02	2.59 ± 0.03	38.58^c^
**16**	0.98 ± 0.01	0.56 ± 0.02	0.41 ± 0.01	42.49^c^
**17**	2.01 ± 0.03	0.95 ± 0.01	1.07 ± 0.04	52.99^c^

^a^Spot number refers to those shown in Fig 1B

^b^ASD ± SD = Average spot density ± Standard deviation

^c^Student’s *t*-test, *p* < 0.05

### Proteomic analysis of *An*. *dissidens* female salivary glands after blood feeding

Salivary gland proteins of age-matched sugar-fed and blood-fed female *An*. *dissidens* were further analyzed using 2-DE. The amount of depletion of each major salivary gland protein as a result of blood feeding was determined by comparison between the two groups (Table 2). Fifteen out of the 17 protein spots showed significant depletion after blood feeding, the exceptions being SN2 and SN12. The amount of depletion in the 15 spots varied from 8.5% for SN6 up to 68.11% for SN5, with a range of values in between for the other 13 spots.

## Discussion

Variation in the physiological states of mosquitoes according to their age, blood feeding status and pathogenic infection have all been shown to be important factors in determining salivary protein amounts and composition [9–15, 25–27]. In the present study it was demonstrated that ageing affected the expression of proteins in the salivary glands of female *An*. *dissidens*. The expression of salivary gland proteins in the mosquitoes varied from 0 to 21 days post emergence. The 17 major salivary gland proteins identified were detectable from day 1 to day 21 post emergence, suggesting that the mosquitoes may be capable of blood feeding within 24 hours of emergence. However, other factors, such as the maturation of the mosquito midgut, and juvenile mosquito hormone levels, are involved in digestive regulation and facilitation [28–32]. Our results are consistent with previous studies in *An*. *barbirostris* species A2 and *An*. *gambiae*. It was shown that the expression of *An*. *barbirostris* species A2 salivary gland proteins is affected by ageing, although the proteome profiles were analyzed only 0 to 60 hours post emergence [12]. The salivary proteome of female *An*. *gambiae* mosquitoes aged 21 days after emergence were shown to be more diverse than the mosquitoes aged 8 days old [11].

From several studies in mosquitoes infected by malaria parasites, *Plasmodium* sporozoites have been discovered in the salivary glands of the mosquito vectors from day 9 to 21 post infection, depending on the species of both parasites and mosquitoes and the ambient temperature [3–6]. In *An*. *dissidens*, they have been found in the salivary glands of the infected mosquitoes at 14 days after infection [18]. For this reason, one purpose of this study was to investigate the salivary proteins that may influence sporozoite maturation using the evidence from the expression patterns of the major salivary gland proteins during adult development. Our results indicate that the proteins in Group 1, which started to show maximum expression in the mosquitoes aged 12 days old or 16 days old onwards, and Group 3, which showed maximum expression on day 12 of adult life, are potentially the most interesting with respect to sporozoite maturation. These proteins include a putative mucin-like protein (SN4), a long form D7 salivary protein (SN10), a putative gVAG protein precursor (SN11), a short form D7r1 salivary protein (SN13), gSG7 salivary proteins (SN15 and 16), an anti-platelet protein (SN8), a short form D7r1 salivary protein (SN12), a D7-related 3.2 protein (SN14), and a gSG6 protein (SN17). These proteins might provide a suitable environment for early stage sporozoites to develop into mature sporozoites. However, most of these proteins including the long form D7, gVAG, short form D7r1, gSG7, anti-platelet protein, D7 related 3, and gSG6, have been characterized as being involved in anti-platelet aggregation, anti-vasoconstriction and anti-inflammatory responses [2, 33–40].

For Group 2, their expression pattern together with the function of apyrase as an anti-platelet aggregation protein [41] and a long form D7 salivary protein (D7L2) that has been shown to reduce blood feeding capacity [38], suggests that apyrase homologues (SN1, 2, and 3), a putative mucin-like protein (SN5), a putative mucin-like protein (SN7), and a long form D7 salivary protein homologue (SN9) may be involved in blood feeding. Regarding a putative mucin-like protein (SN6; Group 4), it was expressed at its highest level on day 3, then steadily falling thereafter suggesting that this protein might not be involved in the maturation of the parasites. Further studies on the roles of these salivary gland proteins involving the regulation of infectivity of sporozoites should be performed.

Choumet and colleagues [42] have recorded activity during the blood-feeding phase of *An*. *gambiae* and shown that young mosquitoes aged 8 days old fed more rapidly than older mosquitoes aged 23 days old. In the *An*. *dissidens* mosquitoes aged 12 to 21 days old, proteins that are homologous to salivary apyrase (SN3), anti-platelet protein (SN8), a long form D7 protein (SN9), D7r1 (SN12), D7 related 3 (SN14), and gSG6 (SN17) were found with decreased in expression on day 12 or 16 of adult life. One possibility is that the decrease in amount of these proteins associated with blood feeding could help in promoting transmission, by providing a longer probing time so giving more opportunity for the sporozoites to enter the skin of a new host, but this requires experimental investigation.

Another purpose of this study was to investigate for proteins that may influence sporozoite transmission, by looking for those that show a high level of depletion immediately after blood feeding. The major proteins which were depleted significantly (more than 30%) after blood feeding in this mosquito species were the homologue proteins to apyrase (SN3), putative mucin-like proteins (SN4, 5 and 7), anti-platelet protein (SN8), long form D7 proteins (SN9 and 10), gVAG protein precursor (SN11), D7 related 3 (SN14), gSG7 (SN15 and 16), and gSG6 (SN17). The results correspond with the findings of the studies into *An*. *gambiae* and *An*. *camprestris*-like [11, 13]. It should also be noted that salivary proteins are not always evenly distributed within the glands and may be present in different amounts in different lobes [20, 43, 44], and this may explain the differential depletion of some proteins compared to others after bloodfeeding.

Researches into sporozoite biology have uncovered the skin stage of the *Plasmodium* life cycle [45–49]. The sporozoites do not directly enter the host bloodstream during a blood meal of mosquitoes. Later they leave the site of injection and find their way to the bloodstream independently [45, 46]. Studies by Amino *et al*. [48] revealed that about half of the sporozoites remain in the skin for up to seven hours, and Yamauchi *et al*. [49] showed that 15–20% of the sporozoites entered the lymphatic system. Matsuoka and colleagues [50] have confirmed that sporozoites can stay in the skin site for more than 42 hours when deposited there by infective mosquitoes. Many sporozoites remain motile for at least 30 minutes at the bite site [47]. Since sporozoites can reside in the skin at the bite sites for hours after injection, the co-inoculation of salivary proteins together with the sporozoites might help in maintaining or supporting optimal conditions for the parasites, before migrating to a blood vessel for passage to the liver or to the draining lymph node.

As discussed above, the results of this study has identified proteins that may have a role in sporozoite maturation and transmission, including the putative mucin-like protein (SN4), the anti-platelet protein (SN8), the long form D7 salivary protein (SN10), the putative gVAG protein precursor (SN11), the D7-related 3.2 protein (SN14), gSG7 salivary proteins (SN15 and 16), and the gSG6 protein (SN17). Studies on infected mosquitoes, including other mosquito species not yet examined, might provide better understanding of the interaction between the salivary proteins identified by depletion following blood feeding, and skin stage sporozoites. Further investigation on the functions of the salivary gland proteins on skin invasion of *Plasmodium* sporozoites could be performed using transient RNA interference (RNAi) gene-silencing assays on the salivary transcribed genes in the mosquito vectors together with the fluorescently labeled *P*. *berghei* parasites [42, 50–52]. Real-time imaging would allow visualization of gliding motility and invasion of the released salivary gland sporozoites at the bite sites and determine probing time and feeding quality and quantity of the mosquitoes.

In conclusion, the changes in the major salivary gland proteins of *An*. *dissidens* during adult development and after blood feeding were analyzed using 2-DE coupled with nanoLC-MS. At least 17 major salivary gland proteins were observed from day one to day 21 post emergence and identified. Fifteen protein spots showed significant depletion after blood feeding with the percentages of the amount of depletion ranging from 8.5% up to 68.11%. The overall results from this study provide candidate proteins that may be involved in sporozoite maturation and transmission, including the putative mucin-like protein, the anti-platelet protein, the long form D7 salivary protein, the putative gVAG protein precursor, the D7-related 3.2 protein, gSG7 salivary proteins, and the gSG6 protein. However, cause and effect cannot be established using data such as these alone, and further studies on their roles in association with maturation and transmission of the malarial parasites, especially interaction between the depleted salivary proteins and skin stage sporozoites should be performed. Comparing these results with our previous work on proteomic analyses of the salivary glands of *An*. *barbirostris* species A2 [12] and *An*. *camprestris*-like [13], members of five major protein families have also been identified as in *An*. *dissidens*, these being apyrase/5′ nucleotidase, anti-platelet protein, long form D7 salivary protein, D7-related protein and gSG6 salivary protein. However, other major proteins with observed molecular masses ranging from 14 to18 kDa and 48 to 56 kDa were either matched with proteins of unknown function or provided no significant match with proteins in the NCBInr database when the search was performed. These may represent salivary proteins with important but as yet undescribed functions in blood feeding, or they may be *An*. *dissidens*-specific proteins, respectively, which may or may not be of particular functional significance. Efficient blood feeding is central to the reproductive success of mosquitoes, so it is not surprising that their salivary glands contain a complex cocktail of proteins with proven or suspected anti-haemostatic or vasodilatory properties. To date a range of different studies on salivary gland proteins have been performed in various mosquito species. Ultimately the value of this study will lie not only in the specific new information relating to *An*. *dissidens*, but as a contribution to future meta-analyses that will hopefully reveal proteins that play roles in malaria transmission, and which could therefore be candidates to target for interruption of such transmission.

## Supporting Information

S1 Fig Three-dimensional views of equivalent regions from representative gels. Arrows indicate HSC70 peaks expressed at different days during adult development.
(TIF)

S1 Table Expression volumes of the 17 major protein spots in the female salivary gland of *An*. *dissidens* determined at different ages in adult developmental time points. (PDF)
