# Supplementary figures and images for: Salivary Gland Proteome during Adult Development and after Blood Feeding of Female Anopheles dissidens Mosquitoes (Diptera: Culicidae)
Source: PLoS One. 2016 Sep 26;11(9):e0163810. doi: 10.1371/journal.pone.0163810 (PMC5036837; doi:10.1371/journal.pone.0163810)

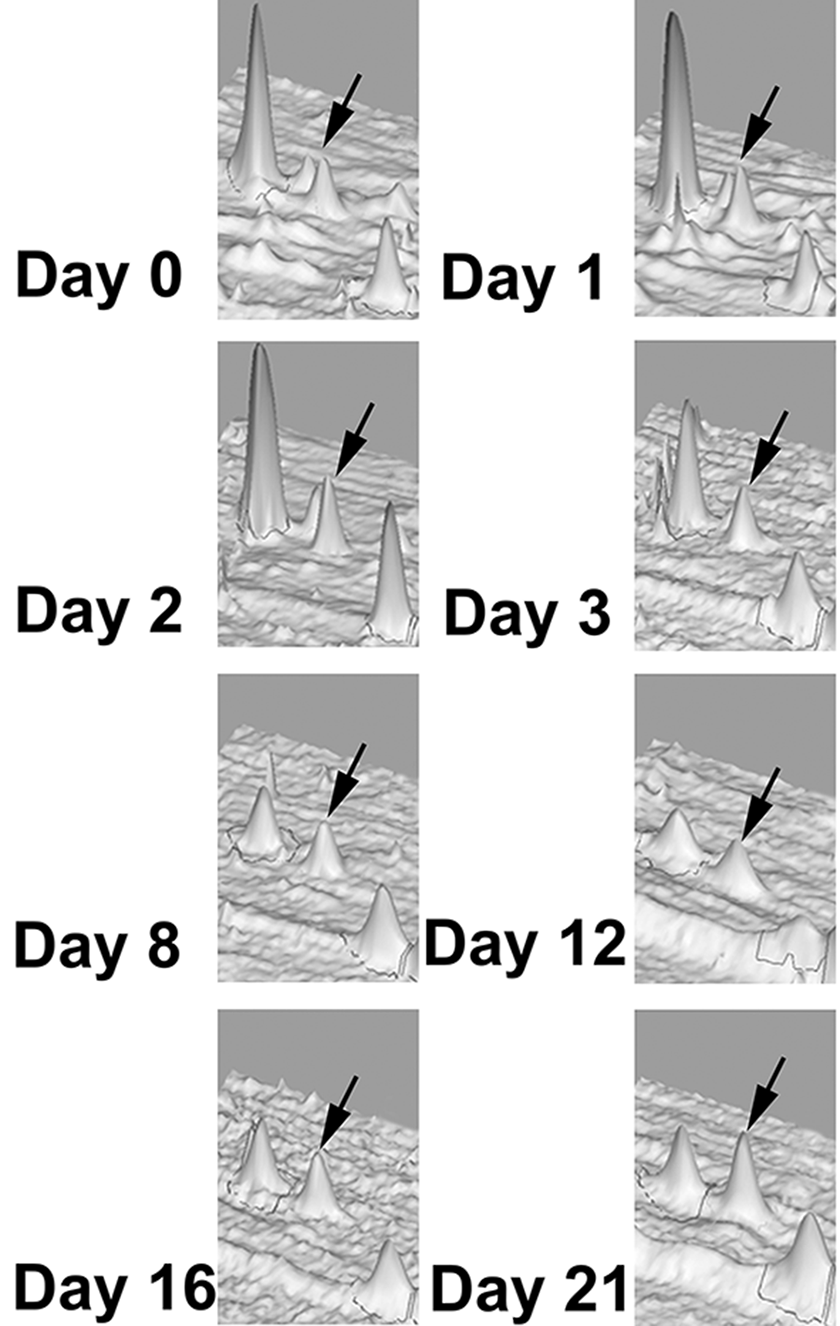

Supplement: S1 Fig — Arrows indicate HSC70 peaks expressed at different days during adult development. (TIF) [file pone.0163810.s001.tif]
