# Supplementary material for: Salivary Gland Proteome during Adult Development and after Blood Feeding of Female Anopheles dissidens Mosquitoes (Diptera: Culicidae)
Source: PLoS One. 2016 Sep 26;11(9):e0163810. doi: 10.1371/journal.pone.0163810 (PMC5036837; doi:10.1371/journal.pone.0163810)
Supplement: S1 Table — (PDF) [file pone.0163810.s002.pdf]

S1 Table. Expression volumes of the 17 major protein spots in the female salivary gland of *An. dissidens* determined at different ages in adult developmental time points.

SN	ASD ± SD							
	**Day0**	**Day1**	**Day2**	**Day3**	**Day8**	**Day12**	**Day16**	**Day21**
**1**	0.00 ± 0.00^{a}	0.07 ± 0.01^{b}	0.22± 0.02^{c}	0.34± 0.02^{c}	0.31± 0.02^{c}	0.32 ± 0.01^{c}	0.33 ± 0.01^{c}	0.32 ± 0.01^{c}
**2**	0.01 ± 0.01^{a}	0.05 ± 0.01^{a}	0.49 ± 0.06^{b}	0.65 ± 0.05^{b}	0.61 ± 0.07^{b}	0.62 ± 0.06^{b}	0.57 ± 0.08^{b}	0.56 ± 0.08^{b}
**3**	0.00 ± 0.00^{a}	0.94 ± 0.02^{b}	3.59 ± 0.05^{c}	3.64 ± 0.02^{c}	3.61 ± 0.05^{c}	3.58 ± 0.06^{c}	3.49 ± 0.05^{d}	1.93 ± 0.04^{e}
**4**	0.00 ± 0.00^{a}	0.27 ± 0.01^{b}	0.50 ± 0.01^{c}	0.57 ± 0.01^{c}	0.88 ± 0.04^{d}	1.41 ± 0.01^{d}	1.38 ± 0.03^{d}	1.37 ± 0.02^{g}
**5**	0.00 ± 0.00^{a}	0.53 ± 0.01^{b}	0.99 ± 0.01^{c}	1.01 ± 0.01^{c}	1.00 ± 0.01^{c}	0.99 ± 0.01^{c}	0.98 ± 0.02^{c}	0.98 ± 0.03^{c}
**6**	0.00 ± 0.00^{a}	0.67 ± 0.01^{b}	1.35 ± 0.02^{c}	1.49 ± 0.05^{c}	1.05 ± 0.06^{b}	1.10 ± 0.04^{b}	0.64 ± 0.01^{b}	0.70 ± 0.05^{b}
**7**	0.24 ± 0.04^{a}	5.67 ± 0.02^{b}	8.61 ± 0.02^{c}	8.77 ± 0.01^{c}	8.62 ± 0.05^{c}	8.23 ± 0.01^{d}	8.22 ± 0.01^{d}	8.24 ± 0.01^{d}
**8**	0.02 ± 0.01^{a}	5.26 ± 0.04^{b}	5.25 ± 0.01^{b}	5.37 ± 0.01^{b}	7.83 ± 0.01^{c}	14.62 ± 0.29^{d}	11.97 ± 0.61^{e}	11.73 ± 0.18^{e}
**9**	0.05 ± 0.01^{a}	1.19 ± 0.02^{b}	2.48 ± 0.07^{c}	3.11 ± 0.01^{d}	3.15 ± 0.05^{d}	3.52 ± 0.04^{e}	3.69 ± 0.04^{f}	3.56 ± 0.04^{e}
**10**	0.04 ± 0.02^{a}	0.75 ± 0.01^{b}	1.05 ± 0.02^{c}	2.15 ± 0.02^{d}	2.85 ± 0.07^{d}	2.92 ± 0.04^{d}	3.78 ± 0.01^{e}	3.77 ± 0.04^{e}
**11**	0.15 ± 0.01^{a}	2.77 ± 0.02^{b}	5.44 ± 0.04^{c}	5.46 ± 0.02^{c}	5.52 ± 0.02^{c}	7.28 ± 0.01^{d}	7.28 ± 0.02^{d}	7.41 ± 0.03^{e}
**12**	0.00 ± 0.00^{a}	0.94 ± 0.02^{b}	1.05 ± 0.06^{b}	1.04 ± 0.07^{b}	1.11 ± 0.05^{b}	4.65 ± 0.04^{c}	3.49 ± 0.05^{d}	3.33 ± 0.06^{e}

**13**	0.27 ± 0.03^{a}	4.01 ± 0.04^{b}	7.75 ± 0.07^{c}	7.89 ± 0.02^{c}	7.88 ± 0.03^{c}	12.24 ± 0.07^{d}	12.43 ± 0.06^{e}	12.22 ± 0.04^{d}
**14**	0.00 ± 0.00^{a}	0.28 ± 0.01^{b}	0.33 ± 0.06^{b}	0.46 ± 0.03^{c}	0.46 ± 0.03^{c}	2.49 ± 0.04^{d}	1.81 ± 0.04^{e}	1.24 ± 0.05^{f}
**15**	0.03 ± 0.01^{a}	2.94 ± 0.04^{b}	5.16 ± 0.06^{c}	5.18 ± 0.05^{c}	7.68 ± 0.05^{d}	7.70 ± 0.04^{d}	7.71 ± 0.01^{d}	10.24 ± 0.04^{e}
**16**	0.00 ± 0.00^{a}	0.46 ± 0.02^{b}	0.70 ± 0.03^{c}	0.70 ± 0.03^{c}	0.70 ± 0.03^{c}	1.01 ± 0.08^{d}	1.03 ± 0.04^{d}	1.01 ± 0.02^{d}
**17**	0.00 ± 0.00^{a}	1.14 ± 0.05^{b}	1.49 ± 0.06^{c}	1.55 ± 0.05^{c}	1.59 ± 0.07^{c}	1.84 ± 0.02^{d}	1.54 ± 0.03^{c}	1.18 ± 0.05^{b}

SN = Spot number refers to those shown in Fig 1B

ASD ± SD = Average spot density ± Standard deviation

Averages followed by different superscript (a, b, c, d, e, f) indicate significant difference ($p < 0.05$).
